# 

**Published:** 1891-01

**Authors:** 


### CONTENTS:

Article I.—A Note Concerning the Action of Chloroform. By Dudley Wilmot Buxton, M.D., B.S. - - 385

" II.—Treatment of Abscesses. By Dr. Frank Overholser. - - - - - - 394

" III.—The Effects of Mercury on the Teeth. By E. D. Mapother, M.D., F. R. C. S. I. - - 417

" IV.—Tannic Acid : Its Internal Administration for Hæmorrhage after Tooth Extraction. By Dr. W. L. Roberts, - - - - 422

### EDITORIAL.

Antiquity of Dentistry. - - - - - - 426

A Cause of Dental Caries. - - - - - - 426

### BIBLIOGRAPHICAL.

A Treatise on the Irregularity of the Teeth and their Correction. 427

The Physicians Visiting List for 1891. - - - - - 429

The Medical Bulletin Visiting List or Physician's Call Record. 429

### MONTHLY SUMMARY.

Art and Dentistry. - - - - - - - - 430

Later Opinions on the Value of Koch's Discovery. - - 431

Retaining Points. - - - - - - - - 432

